# Private parts for private property: evolution of penis size with more valuable, easily stolen shells

**DOI:** 10.1098/rsos.181760

**Published:** 2019-01-16

**Authors:** Mark E. Laidre

**Affiliations:** Department of Biological Sciences, Dartmouth College, Hanover, NH 03755, USA

**Keywords:** comparative analyses, conflict and cooperation, private property, penis size, shells, social evolution

## Abstract

Evolution has generated enormous diversity in animal genitalia. However, the importance of private property in driving penis size evolution has rarely been explored. Here, I introduce a novel hypothesis, the ‘private parts for private property' hypothesis, which posits that enlarged penises evolved to prevent the theft of property during sex. I tested this hypothesis in hermit crabs, which carry valuable portable property (a shell) and which must emerge from this shell during sex, risking social theft of their property by eavesdroppers. I measured relative penis size (penis-to-body ratio) for *N* = 328 specimens spanning nine closely related species. Species carrying more valuable, more easily stolen property had significantly larger penis size than species carrying less valuable, less easily stolen property, which, in turn, had larger penis size than species carrying no property at all. These patterns in penis size remained even when phylogeny was controlled for, and the patterns were not explained by alternative hypotheses. Instead, the results suggest larger penises evolved as morphological adaptations to facilitate safe sex, in which individuals retain their valuable property by extending a long penis outside the shell to copulate. This hypothesis may likewise apply to other taxa, including those with valuable but non-portable property.

## 1. Introduction

In some species, selection has favoured the evolution of large penises [1], which may maximize male reproductive success through various means (e.g. acting as a display favoured by female choice, physically stimulating females, overcoming rival sperm competition or bypassing female defences [2,3]). Perhaps the earliest hypothesis for the evolution of large penis size was posited by Darwin, who discovered that barnacles have the biggest penis size in the animal kingdom relative to body size. Of the barnacle penis, Darwin remarked [4], it ‘is wonderfully developed, so that in *Cryptophialus*, when fully extended, it must equal between eight and nine times the entire length of the animal!' (p. 26). Darwin conjectured that these long penises evolved to enable insemination of distant neighbours, each physically rooted at separate locations (see also [5] for an alternative solution involving sperm casting).

Here, I examine a novel hypothesis for the evolution of large penis size, likewise inspired by crustaceans and further extending Darwin's original insight that long penises may evolve as specialized adaptations, which overcome ecological challenges via increased sexual reach. This new hypothesis builds upon the fact that many animals use private property [6], which exists independent of and external to their body, must be secured and defended against conspecifics, and may be put in heightened danger of theft, particularly while individuals are preoccupied during sex. Paralleling Darwin's hypothesis that long barnacle penises evolved to facilitate sex with distant neighbours, I suggest that some animals might evolve long penises to protect their private property from being stolen during sex. In theory, longer penises could enable individuals to reach out to sexual partners while simultaneously maintaining a safe grip on their property with the rest of their body, thus safeguarding property against thieves while having sex.

Many species must negotiate ownership of private property, immediately before and during copulation (e.g. food and other nuptial gifts, territories, extra-seminal fluids; see [6] for a review of examples, from microbes to vertebrates). The ‘private parts for private property' hypothesis is especially applicable in these contexts, potentially generalizing across a diversity of species in which property theft is possible. However, with respect to portable private property, arguably no more clear-cut example exists among non-human animals than that of hermit crabs [7], which obligately carry a transportable shell as a form of individual property, one that is separate from their body but is essential to their survival as an external shelter [8]. Hermit crabs thus offer a model system for testing the ‘private parts for private property' hypothesis. Indeed, copulation between hermit crabs, while not involving internal fertilization, nevertheless involves ‘inside the shell’ fertilization, in which pairs orient their shell apertures face-to-face and males transfer their spermatophore by ejaculating on the outside of the female's body but into her shell [9]. Critically, these sexual interactions require that individuals come at least partway out of their shells [9], thus presenting an inherent danger that one's private property—the shell itself—might be stolen in the midst of mating. Theft may occur either by one's current sexual partner or, more commonly, by third-party conspecific ‘intruders' [9,10], which socially eavesdrop [11] and can evict others [12], targeting sexual pairs as they are mating and sometimes successfully stealing their property (Laidre, personal observation in *Coenobita* spp.).

The risk of property theft during sex is differentially high in some species of hermit crab, which architecturally modify shells [13–16], rendering these shells more valuable and more easily stolen private property [12,17,18]. Specifically, unlike marine hermit crabs [19], terrestrial hermit crabs (*Coenobita* spp.) remodel the interior of shells and these costly construction efforts [20] make remodelled shells highly preferred and more valuable resources. Indeed, while resource value differences exist in marine hermit crabs (which prefer certain shell species over others), the magnitude of such differences is dwarfed by the difference in resource value between remodelled versus unremodelled shells in terrestrial hermit crabs: without a remodelled shell, adult terrestrial hermit crabs will desiccate and die within 24 h, even if given an unremodelled shell in exchange [17]. Losing one's remodelled shell can thus be perilous. Furthermore, remodelled shells can be more easily stolen than unremodelled shells: this is because the internal columella (upon which hermit crabs maintain their abdominal grip and thereby avoid eviction [12,18]) is eroded away in remodelled shells [13–16], meaning owners of such shells can have their property snatched from them more readily [12,17,18]. Individuals thus face competing fitness demands: on the one hand, seeking to mate and pass on their genes, on the other hand, facing potential death if their private property is stolen from them during sex. An adaptive solution to these conflicting pressures could be mediated through penis size: larger, more elongated penises could enable individuals to engage in protected sex, in which a male extends a long penis outside of his shell and into a female's shell, while each member of the couple keeps the rest of their body safely inside their shell, thereby retaining hold of their valuable private property.

Here, I test this ‘private parts for private property' hypothesis, which posits that enlarged penises evolved to prevent the theft of valuable private property during risky sexual liaisons. To test this hypothesis, I compared penis size across hermit crabs, specifically contrasting species that (1) carry more valuable, more easily stolen property (remodelled shells) against (2) their closest evolutionary relatives that either (a) carry less valuable, less easily stolen property (unremodelled shells) or (b) do not carry property at all. Critically, the ‘private parts for private property' hypothesis predicts that large penis size relative to body size will be most likely to evolve in the former species, which face the greatest risk of their valuable external property being stolen during sex.

## 2. Material and methods

### 2.1. Species comparisons

I used the molecular phylogeny of hermit crabs [21] (Crustacea, Decapoda, Anomura) to contrast penis size between closely related species that use different types of private property or no private property at all. I focused on species from the genus *Coenobita*, which use remodelled shells, unlike all other species of hermit crab [13,22]. Three *Coenobita* species (*C. clypeatus*, *C. compressus* and *C. perlatus*) have been mapped onto this phylogeny, so I compared these species to their closest phylogenetic relatives [21], which either (i) branched off ancestrally and use unremodelled shells (*Isocheles pilosus*, *Clibanarius albidigitus*, *Calcinus obscurus*, *Petrochirus diogenes* and *Dardanus insignis*) or (ii) are more highly derived, and use remodelled shells only as juveniles and then cease using shells before sexual maturity (*Birgus latro*) [23]. All species were measured from museum specimens preserved in ethanol at the Smithsonian Institution in Washington DC and the Harvard Museum of Comparative Zoology (MCZ) in Cambridge, MA. I exhaustively sought species from every genus that surrounds *Coenobita* as a branch on the phylogenetic tree (fig. 1 in [21], reproduced with permission as electronic supplementary material, figure S1 in the present paper). I measured a total of *N* = 328 specimens, representing nine species, seven genera and two families (Coenobitidae and Diogenidae). Sample size for each species depended on available specimens in the collections. A minimum of *N* = 19 specimens were measured for every species (see electronic supplementary material, table S1 for sample sizes; all data are available as electronic supplementary material).

### 2.2. Standardized measurements

**Figure 1.**
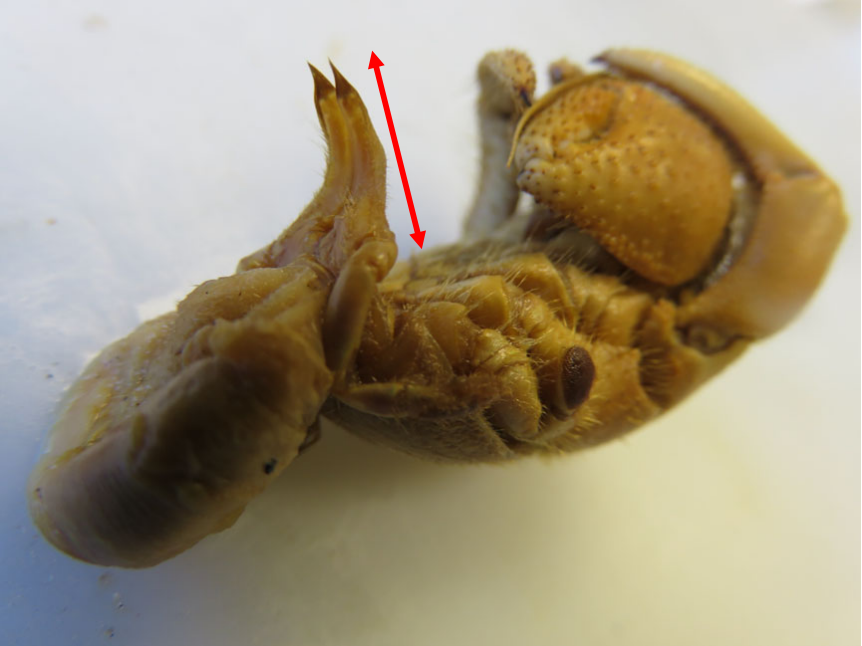
Penis of male hermit crab. The male is from the highly social terrestrial species (*Coenobita compressus*), which architecturally remodels shells, thereby making these shells more valuable and more easily stolen private property. The male is naked (outside of his shell), and the red arrow designates the length of the male's ‘penis' or ‘sexual tubes'. To the right are the male's anterior appendages (claws and walking legs) and to the left is the male's abdomen, which usually grips onto a shell—his private property. Photo by the author.

Specimens were measured with electronic callipers to the nearest 0.01 mm. To compare relative penis size across species, a penis size to body size ratio was calculated based on two measures taken on every specimen: (i) shield length (a metric of body size) and (ii) fifth coxae length (a metric of penis size) (figure 1; electronic supplementary material, figure S2). In males, fifth coxae length represents the size of the ‘private parts', which in some species are elongated into tubular, hardened protrusions referred to as sexual tubes [9,10,24], which function as a penis and contain the ejaculatory ducts to transfer sperm during copulation.^1^ In females,^2^ by contrast, the fifth coxae serve no sexual or reproductive function. The size of this structure in females therefore provided a useful reference point within each species, indicating the extent to which male genitals have diverged from baseline. I thus measured the length of the fifth coxae in females as a relevant control, which together with shield length allowed female coxae-to-body ratio to be compared with males, revealing sexual dimorphism in the size of this structure across species.

### 2.3. Multiple hypotheses and predictions

The ‘private parts for private property' hypothesis predicts that large penis size relative to body size will occur in species that carry more valuable, more easily stolen property. *Coenobita* species, which carry remodelled shells, therefore should have significantly larger relative penis size than closely related species that carry unremodelled shells. Additionally, species that carry unremodelled shells (while facing reduced risks of property theft during sex) still face more risk than species that carry no property at all, because the latter, by definition, have zero risk of property theft. Hence, species with unremodelled shells should have significantly larger relative penis size than species that carry no property. Finally, the ‘private parts for private property' hypothesis also predicts that higher levels of shell remodelling (*C. compressus* is known to perform the most remodelling, *C. clypeatus* the least and *C. perlatus* an intermediate amount [13–20,22]) should be positively correlated with longer penis size, because a greater degree of remodelling makes shells even more valuable and even more easily stolen.

A set of alternative hypotheses, each with unique predictions that differed from the above hypothesis, were also tested:

(1) ‘Carrying anything’: if carrying anything (irrespective of its value as private property or the ease with which it can be stolen) favours the evolution of large penises, then species carrying unremodelled shells should have penises just as large as species carrying remodelled shells.
(2) ‘Constraints of living inside versus outside shells’: if living inside a shell constrains the evolution of large penis size, while living outside a shell removes these constraints, then *Birgus latro* (which ceases using a shell very early in its development and long before it reaches sexual maturity [23]) should have the largest relative penis size of all.
(3) ‘By-product of body size’: if simply attaining bigger body size somehow favours the evolution of proportionately larger penis size, then *Petrochirus diogenes* (known as the ‘giant marine hermit crab’ and one of the biggest deep sea crustaceans [21]) and *Birgus latro* (the world's largest terrestrial invertebrate [23]) should both have the largest relative penis sizes. Conversely, if small body size has this effect, then the smallest bodied species (*Isocheles pilosus*, *Clibanarius albidigitus* and *Calcinus obscurus*) should instead have the largest relative penis sizes.
(4) ‘Environment, not property’: if the general surrounding environment (living in the sea versus on land) favours the evolution of large penis size, independent of private property, then either all terrestrial hermit crabs (*Birgus latro* and *Coenobita* spp.) or all marine hermit crabs (the rest of the species) should have larger relative penis size.

### 2.4. Statistical analyses

The above hypotheses were tested by comparing male penis-to-body ratio across species. A first set of statistical analyses were performed in JMP^®^ Pro 12.1.0 using a one-way ANOVA, along with orthogonal contrast tests. This initial analysis did not control for phylogeny, but rather used all individual specimen measures within each species as data points (see raw Excel data file in electronic supplementary material). This analysis thus enabled single species to be contrasted against other species, based on the variation between versus within each species, following a standard ANOVA.

A second statistical analysis then used phylogenetically controlled comparative methods to test whether the key result from the first set of analyses still remained robust once shared ancestry within the tree was explicitly controlled for (using the established molecular phylogeny and its known branch lengths [21]). The aim of this second analysis was thus to test whether potential correlations among values between species, due simply to common ancestry, could be excluded as a driving explanation. This phylogenetically controlled analysis was performed in the nlme package in R using generalized least-squares (GLS), with ML lambda used in the phylogenetic autocorrelation matrix (see [25,26] on the mathematical foundations behind this approach). For this analysis, each of the three different shell-type categories (remodelled, unremodelled and no property at all) was included, and then a phylogenetically controlled contrast was carried out to compare male penis-to-body ratio between (i) species which carry remodelled shells versus (ii) species which carry unremodelled shells. In the Results section, this analysis is designated ‘phylogenetically controlled contrast' to differentiate it from the other contrasts, which were not phylogenetically controlled. (Note, phylogenetically controlled contrasts could not be conducted to compare the one species which carries no property versus other species, because these phylogenetically controlled methods consolidate all measures of individuals within a species to just a single mean value for the species.)

Finally, for within-species tests, I compared male versus female coxae-to-body ratio in each species using two-sample *t*-tests. For all analyses described here, the overall alpha level was controlled at 0.05 and the Bonferroni method was used when undertaking multiple tests.

## 3. Results

**Figure 2.**
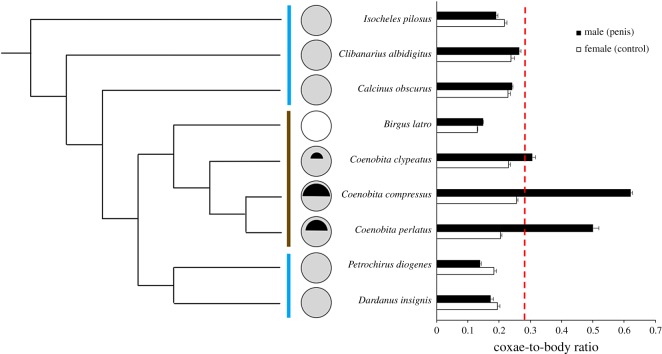
Longer penises evolved in species with more valuable, more easily stolen private property. Coxae-to-body ratio measured in males and females (for males indicates relative penis size and for females indicates a control) across nine closely related hermit crab species arrayed based on phylogeny [21]. Blue line = marine; brown line = terrestrial. Circles designate the type of private property (white circle = no shell; grey circle = unremodelled shell; with black half-circle = remodelled shell, and with bigger black half-circles indicating a relatively greater level of remodelling in those species, which makes the shells even more valuable and more easily stolen). Dashed red line separates *Coenobita* species, which carry more valuable and more easily stolen private property (i.e. remodelled shells), from all other species.

**Figure 3.**
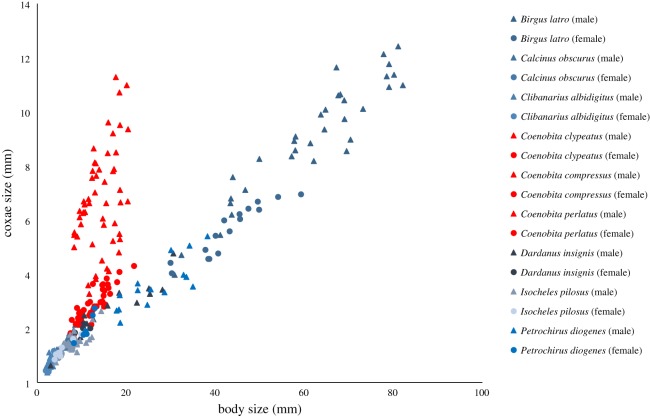
Penis to body size relationship in male *Coenobita* differs from all other species. Coxae size and body size measured in males and females (indicating penis size and a control, respectively) across all nine closely related hermit crab species. Triangles = males. Circles = females. Red = the three *Coenobita* species. Blue = all other species (each in a different shade of blue).

Male penis-to-body ratio varied significantly across species (one-way ANOVA: *F*_8,210_ = 388.98, *p* < 0.0001). Whereas none of the four alternative hypotheses were supported, the ‘private parts for private property' hypothesis was supported (figure 2). In particular, *Coenobita* species had significantly larger penises relative to their bodies than all other species (contrast test: *F*_1,210_ = 2092.96, *p* < 0.0001). Indeed, for over an order of magnitude difference in body size across species, the relationship between penis size and body size remained nearly constant, differing only in males of *Coenobita* spp. (figure 3). Moreover, *Coenobita* species, which carry remodelled shells, had significantly larger relative penis size than species which carry unremodelled shells (contrast test: *F*_1,210_ = 1823.43, *p* < 0.0001). And species which carry unremodelled shells, in turn, had significantly larger relative penis size than the species which carries no property at all (contrast test: *F*_1,210_ = 48.75, *p* < 0.0001). Given the small total number of species (*N* = 9) and their shared ancestry, it was noteworthy that the phylogenetically controlled analysis strongly supported the above results: a significant difference existed in male penis-to-body ratio when species which carry remodelled shells were contrasted with species which carry unremodelled shells (phylogenetically controlled contrast: *F*_2,6_ = 13.74, *p* = 0.0058).

Interestingly, variation within the *Coenobita* genus further supported the ‘private parts for private property' hypothesis. *C. compressus*, which performs the most remodelling, had the biggest penis, significantly exceeding that of *C. perlatus*, which performs an intermediate level of remodelling (contrast test: *F*_1,210_ = 93.41, *p* < 0.0001); and *C. perlatus*, in turn, had a significantly larger penis than *C. clypeatus*, which performs the least remodelling and which had the smallest penis among the three (contrast test: *F*_1,210_ = 218.61, *p* < 0.0001). Comparisons of males and females within each *Coenobita* species revealed that the male penis was substantially enlarged relative to the equivalent non-reproductive structure in females, with males exceeding conspecific females in coxae-to-body ratio by over two times (*t*-test of males versus females for *C. compressus*: *t* = 41.99, d.f. = 32.21, *p* < 0.0001; *C. perlatus*: *t* = 15.15, d.f. = 18.75, *p* < 0.0001; *C. clypeatus*: *t* = 5.78, d.f. = 22.84, *p* < 0.0001). By contrast, within every non-*Coenobita* species there was either (i) no significant difference between males and females in coxae-to-body ratio (*t*-test for *Calcinus*: *t* = 1.52, d.f. = 14.89, *p* = 0.15; *Clibanarius*: *t* = 1.90, d.f. = 23.45, *p* = 0.069; *Dardanus*: *t* = 1.74, d.f. = 15.97, *p* = 0.10). Or (ii) females actually exceeded males in coxal extension (*t*-test for *Isocheles*: *t* = 2.95, d.f. = 16.10, *p* = 0.0093; *Petrochirus*: *t* = 4.68, d.f. = 10.12, *p* = 0.0008). Or (iii) males exceeded females but only by a minute fraction (*t*-test for *Birgus*: *t* = 5.60, d.f. = 38.61, *p* < 0.0001).

## 4. Discussion

Conflicts during sex can occur due to divergent evolutionary interests between males and females [2,27]. These dyadic sexual conflicts, however, can also be embedded in broader polyadic social conflicts [6], in which a graver danger may be posed by third-party conspecifics that eavesdrop on mating pairs and seek to steal their private property while they are sexually engaged. The present study suggests a novel solution for protecting private property from being stolen during sex: evolving larger penises. These enlarged ‘private parts' then become ‘public parts', as they are what gets exposed to the outside world during sex, while the rest of the body remains firmly tucked inside the shell. For species with more valuable and more easily stolen property, a longer penis can thus extend out of a male's shell and into a female's shell, allowing both parties to maintain more secure, decisive grips on their property by not coming out as far of their shells. The evolutionary pattern of relative penis size across species is consistent with this ‘private parts for private property' hypothesis, especially the intense level of social competition for remodelled shells that exists within the housing markets of *Coenobita* species [9–20,22]. Critically, all other hypotheses for these penis size patterns came up short. Moreover, the additional space available within remodelled shells cannot be interpreted as an evolutionary cause of enlarged penis size: marine hermit crabs have ample excess room inside their unremodelled shells [13], which would allow them to easily accommodate much larger penis sizes than they have; and even coconut crabs, which have no space constraints [23], nevertheless have miniscule relative penis size.

The morphological adaptation of longer penis size may be complemented by behavioural adaptations, which taken together could provide added ‘insurance' that private property is not stolen [6]. Indeed, the threat posed by conspecific eavesdroppers should place a strong premium on both speedy and secretive sex, particularly in species with more valuable and more easily stolen private property. Supporting this, the mating duration for *Coenobita* spp. is reportedly faster [9], often lasting just a few minutes [10], compared to much longer mating times in marine hermit crabs, which carry less valuable and less easily stolen shells. Moreover, given that *Coenobita* spp. frequently roam open beachfront areas during daylight and have keen eyesight for visually detecting conspecific associations from a distance [11,28], it is notable that individuals instead opt to have sex at night within secluded caves [10]. These secretive ‘quickies' may behaviourally and temporally reinforce the morphology of a long penis, better ensuring pairs can evade eavesdroppers and thus property theft.

The species with the longest penis-to-body size ratio in this study, *Coenobita compressus*, has been one of the best-studied hermit crabs in the wild, with respect to sex [9,10] and its complex social life [18,29,30], which revolves around architecturally remodelled shells [11–20,22,28–30]. Surprisingly though, neither male behaviour nor male body size in *C. compressus* has been found to predict mating success [9,10], defined as successful spermatophore transfer to females. The results of the present study suggest that intra-specific variation in relative penis size could perhaps be a key determinant of mating success: such variation is considerable (despite even greater interspecific variation), so males with relatively longer penises for their body size might, therefore, be able to insert their sexual tubes deeper inside females' shells, succeeding more often in transferring spermatophores. In addition to exploring the consequences of intra-specific variation, further work on inter-specific variation in penises would be valuable. For example, morphological variation between *Coenobita* species includes penises bent at various angles [9,24], which in theory could adaptively permit males of some species to simultaneous mate and fight (e.g. inserting their penis into female shells on one side, while driving off property thieves and rival males on their other side). Worldwide, approximately 20 *Coenobita* species exist [22], and while all are known to use remodelled shells [13], no study has quantified the relative level of shell remodelling across these species or mapped any more than the three species examined here onto a molecular phylogeny. A robust phylogeny of *Coenobita*, alongside precise measures of shell remodelling across all these species, could enable further tests of the evolution of penis size and its relation to private property, as well as to social evolution more broadly [18,20,30]. Intriguingly, distant relatives in Paguridae (separate from Coenobitidae and Diogenidae) also independently evolved sexual tubes [9,31]; though the functions of these tubes, many of which are bendable rather than permanently hardened, remain mysterious due to a lack of observations on how they are used in nature.

The ‘private parts for private property' hypothesis provides a novel explanation for the evolution of large penis size. This hypothesis may also inform general tests of private property that shed light on an opposite trend: the evolution of small relative penis size. Phenotypic investments in sexual structures (e.g. penis size) probably trade off against fighting structures (e.g. claw size), with one or the other of these structures being more effective in retaining ownership over property depending on the circumstances [6,32,33]. For animals with private property that cannot be carried, and is instead embodied by larger territories and landscapes, the only way to retain a ‘grip' on such property is by investing in resource-holding potential, as represented by a property owner's body size, fighting ability and weaponry [34]. Such investments, by trading off against penis size, may create alternative axes of property defence: weapon size versus penis size [32,33]. In the present study, the giant coconut crab (*Birgus latro*) had a meagre relative penis size, among the very smallest of all species measured; this species instead puts extreme investment into powerful claws [23], which enable predation and defence of an extensive non-portable territory and burrow system [20,35]. Broader comparative analyses across taxa could thus explore such trade-offs by examining relative weapon and penis size in relation to private property value.

## Supplementary Material

Table S1;Figure S1;Figure S2

## Supplementary Material

All raw data

## Footnotes

1 For species in which males had an asymmetrical penis, with their two sexual tubes differing in length, I measured the longer of the two tubes, which reflected a male's maximal sexual reach.

2 In all species of hermit crab, males and females can be differentiated as follows: males gonopores are on their fifth periopods, whereas female gonopores are on their third periopods (i.e. at the base of their walking legs). Females release eggs from these gonopores, which are flat holes and which lack any extension or structure in all species measured.

## Data accessibility

The entire dataset is available online in the electronic supplementary material.
