# Supplementary material for: Private parts for private property: evolution of penis size with more valuable, easily stolen shells
Source: R Soc Open Sci. 2019 Jan 16;6(1):181760. doi: 10.1098/rsos.181760 (PMC6366222; doi:10.1098/rsos.181760)
Supplement: Table S1;Figure S1;Figure S2 [file rsos181760supp1.docx]

**ELECTRONIC SUPPLEMENTARY MATERIAL**

**For:**

**Private parts for private property:**

**evolution of penis size with more valuable, easily stolen shells**

Mark E. Laidre

***Royal Society Open Science***

**Table S1.** (below)

**Supplementary Figure Legends**

**Figure S1.** Section of molecular phylogeny of hermit crabs (taken from [21] with permission from the corresponding author Heather Bracken-Grissom).

**Figure S2.** Caliper measurements of (**A**) fifth coxae length (a metric of penis size) and (**B**) shield length (a metric of body size) in a *Coenobita compressus* male.

**Table S1.** Sample size across hermit crab species (N = 328 total specimens).

| **Shell used as private property** | **Species of hermit crab** | **Male** | **Female** | **Total** |
| --- | --- | --- | --- | --- |
| ***Remodeled***  (more valuable, more easily stolen) | *Coenobita clypeatus* | 16 | 15 | 31 |
|  | *Coenobita compressus* | 20 | 20 | 40 |
|  | *Coenobita perlatus* | 18 | 12 | 30 |
| ***Unremodeled***  (less valuable, less easily stolen) | *Isocheles pilosus* | 40 | 9 | 49 |
|  | *Clibanarius albidigitus* | 36 | 13 | 49 |
|  | *Calcinus obscurus* | 28 | 12 | 40 |
|  | *Petrochirus diogenes* | 15 | 6 | 21 |
|  | *Dardanus insignis* | 13 | 6 | 19 |
| ***None***  (cease using shells at sexual maturity) | *Birgus latro* | 33 | 16 | 49 |
|  |  |  |  | N = 328 |


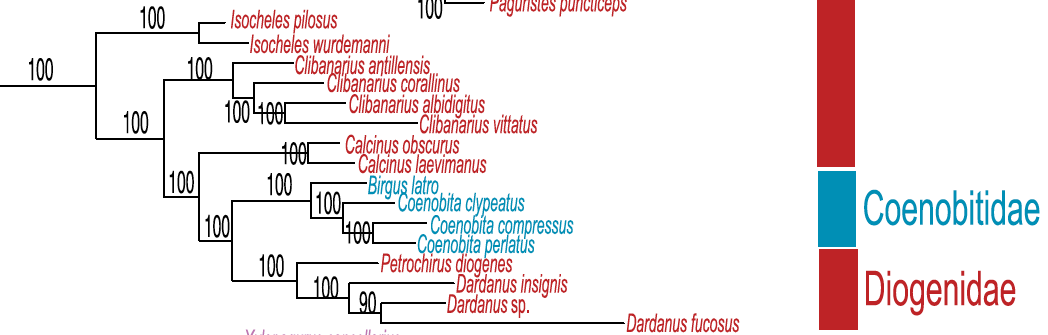


**Figure S1**


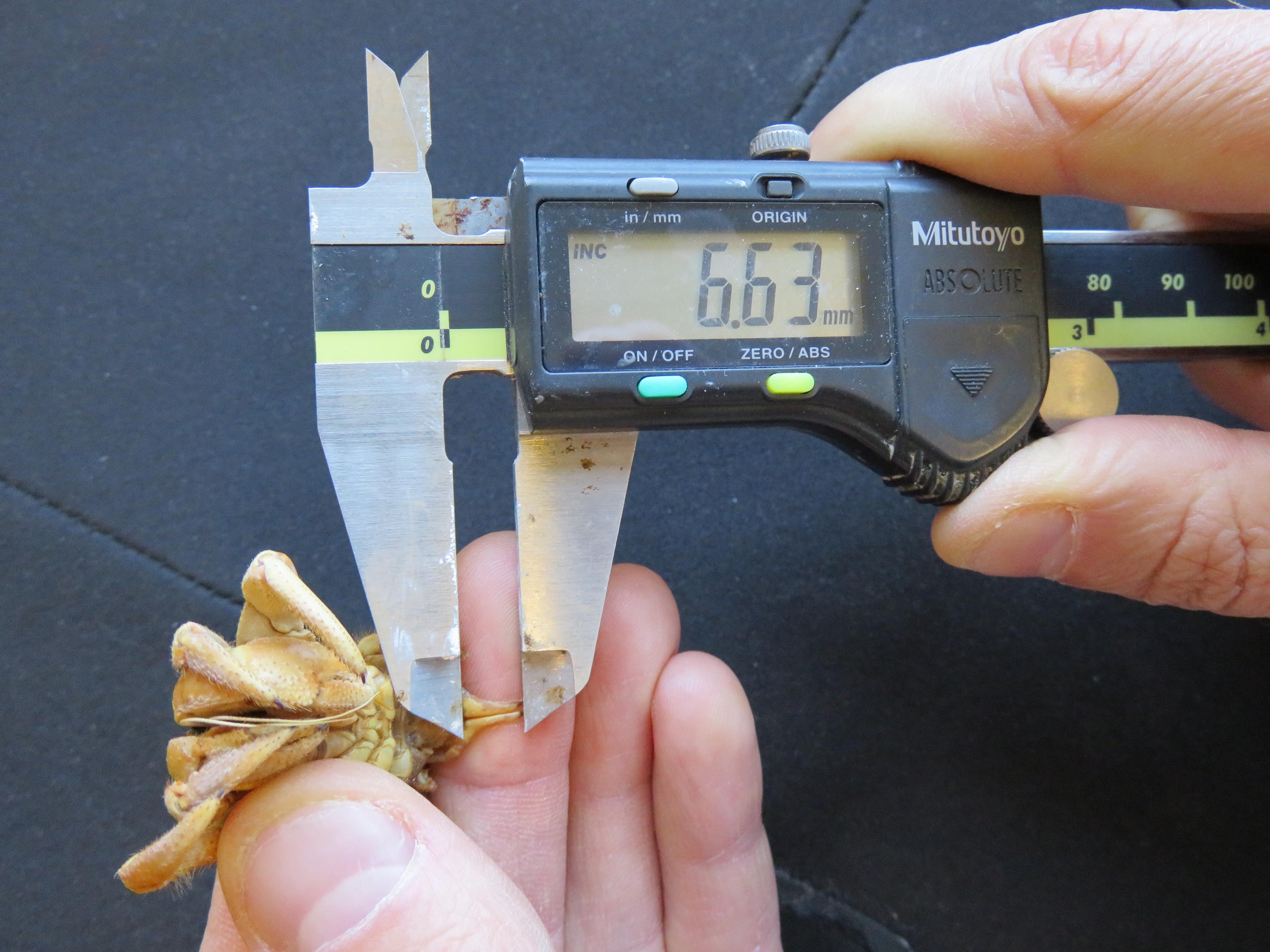

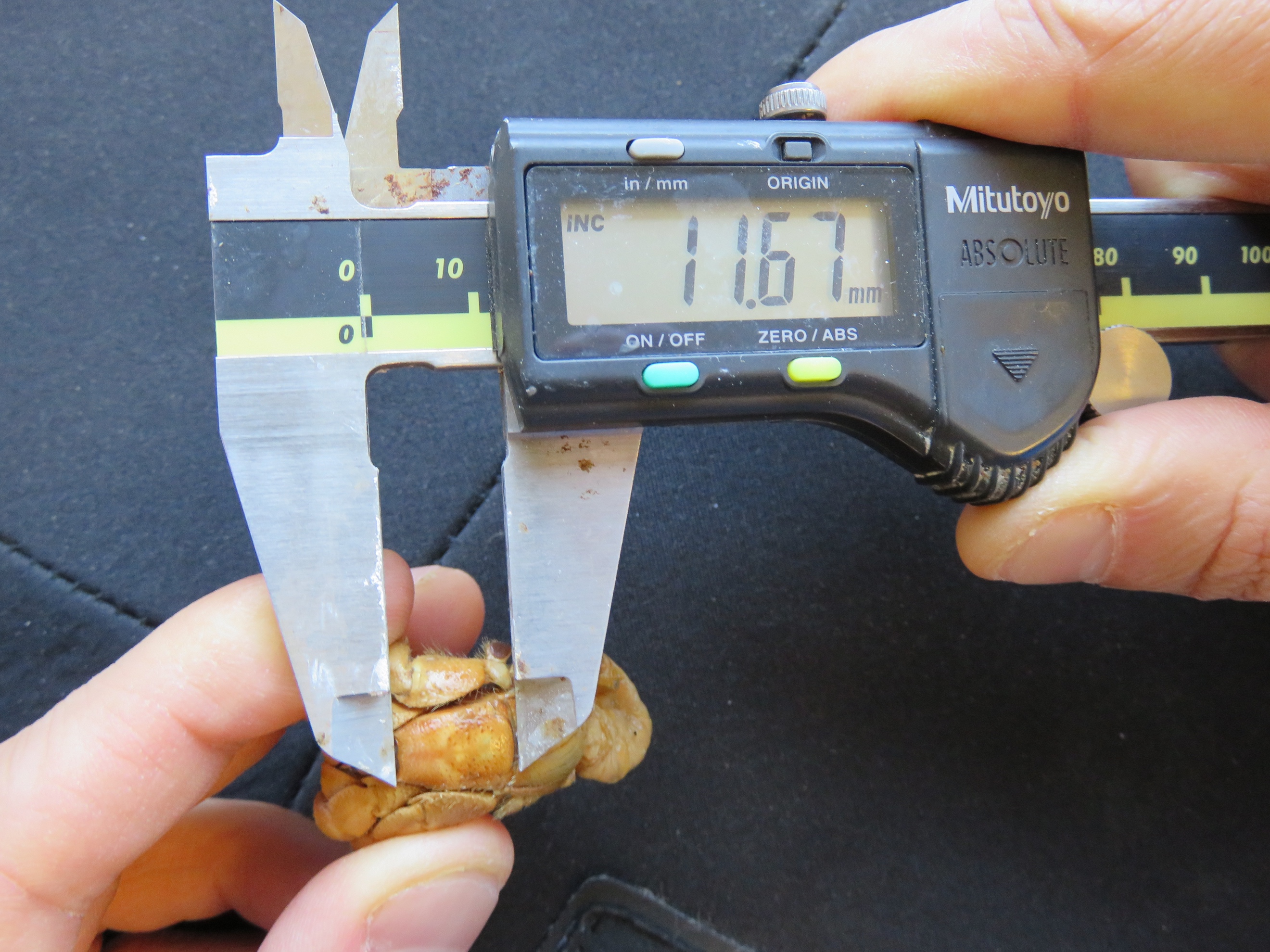


**A**

**B**

**Figure S2**
